# Targeted hypothermia versus targeted normothermia after out-of-hospital cardiac arrest: a statistical analysis plan

**DOI:** 10.1186/s13063-020-04654-y

**Published:** 2020-10-07

**Authors:** Janus Christian Jakobsen, Josef Dankiewicz, Theis Lange, Tobias Cronberg, Gisela Lilja, Helena Levin, Jan Bělohlávek, Clifton Callaway, Alain Cariou, David Erlinge, Jan Hovdenes, Michael Joannidis, Per Nordberg, Mauro Oddo, Paolo Pelosi, Hans Kirkegaard, Glenn Eastwood, Christian Rylander, Manoj Saxena, Christian Storm, Fabio Silvio Taccone, Matthew P. Wise, Matt P. G. Morgan, Paul Young, Alistair Nichol, Hans Friberg, Susann Ullén, Niklas Nielsen

**Affiliations:** 1 Copenhagen Trial Unit, Centre for Clinical Intervention Research, Department 7812, Rigshospitalet, Copenhagen University Hospital, Tagensvej 22, Copenhagen, Denmark; 2 Department of Cardiology, Holbæk Hospital, Holbæk, Denmark; 3 Department of Regional Health Research, The Faculty of Health Sciences, University of Southern Denmark, Odense, Denmark; 4 Department of Clinical Sciences, Cardiology, Lund University, Skåne University Hospital, Lund, Sweden; 5 Section of Biostatistics, Faculty of Health and Medical Sciences, University of Copenhagen, Copenhagen, Denmark; 6 Department of Clinical Sciences, Neurology, Lund University, Skåne University Hospital, Lund, Sweden; 7 Department of Clinical Sciences, Research and Education, Lund University, Skåne University Hospital, Lund, Sweden; 8 2nd Department of Medicine, First Faculty of Medicine, Charles University in Prague and General University Hospital, Prague, Czech Republic; 9 Department of Emergency Medicine, University of Pittsburgh, Pittsburgh, PA USA; 10 Medical Intensive Care Unit, Cochin University Hospital (APHP) and Paris Descartes University, Paris, France; 11 Department of Anesthesia and Intensive Care, Oslo University Hospital, Rikshospitalet, Oslo, Norway; 12 Division of Intensive Care and Emergency Medicine, Department of Internal Medicine, Medical University Innsbruck, Innsbruck, Austria; 13 Department of Medicine, Center for Resuscitation Science, Karolinska Institute, Solna, Sweden; 14 Department of Intensive Care Medicine, Faculty of Biology and Medicine, Centre Hospitalier Universitaire Vaudois (CHUV) - University Hospital, University of Lausanne, Lausanne, Switzerland; 15 Department of Surgical Sciences and Integrated Diagnostics (DISC), University of Genoa, Genoa, Italy; 16 San Martino Policlinico Hospital, IRCCS for Oncology and Neurosciences, University of Genoa, Genoa, Italy; 17 Research Center for Emergency Medicine, Department of Clinical Medicine, Aarhus University Hospital and Aarhus University, Aarhus N, Denmark; 18 Department of Intensive Care, Austin Hospital, Heidelberg, Australia; 19 Department of Anesthesiology and Intensive Care Medicine, Institute of Clinical Sciences, Sahlgrenska Academy, University of Gothenburg, Gothenburg, Sweden; 20 Bankstown Hospital Clinical School and The George Institute for Global Health, University of New South Wales, Kensington, Australia; 21 Department of Nephrology and Medical Intensive Care, Charité - Universitätsmedizin Berlin, Berlin, Germany; 22 Division of Neuroscience Critical Care, Department of Anesthesiology and Critical Care Medicine, Johns Hopkins University School of Medicine, Baltimore, MD USA; 23 Department of Intensive Care, Erasme University Hospital, Université Libre de Bruxelles (ULB), Brussels, Belgium; 24 Adult Critical Care, University Hospital of Wales, Cardiff, UK; 25 Medical Research Institute of New Zealand, Wellington, New Zealand; 26 University Collage Dublin-Clinical Research Centre, St Vincent’s University Hospital, Dublin, Ireland; 27 Australian and New Zealand Intensive Care-Research Centre, Monash University, Melbourne, Australia; 28 Department of Critical Care, Alfred Hospital, Melbourne, Australia; 29 Department of Clinical Sciences, Anesthesia & Intensive Care, Lund University, Skåne University Hospital, Lund, Sweden; 30 Clinical Studies Sweden, Skåne University Hospital, Lund, Sweden; 31 Department of Clinical Sciences Lund, Anesthesia & Intensive Care, Lund University, Helsingborg Hospital, Lund, Sweden

## Abstract

**Background:**

To date, targeted temperature management (TTM) is the only neuroprotective intervention after resuscitation from cardiac arrest that is recommended by guidelines. The evidence on the effects of TTM is unclear.

**Methods/design:**

The Targeted Hypothermia Versus Targeted Normothermia After Out-of-hospital Cardiac Arrest (TTM2) trial is an international, multicentre, parallel group, investigator-initiated, randomised, superiority trial in which TTM with a target temperature of 33 °C after cardiac arrest will be compared with a strategy to maintain normothermia and active treatment of fever (≥ 37.8 °C). Prognosticators, outcome assessors, the steering group, the trial coordinating team, and trial statisticians will be blinded to treatment allocation. The primary outcome will be all-cause mortality at 180 days after randomisation. We estimate a 55% mortality in the targeted normothermia group. To detect an absolute risk reduction of 7.5% with an alpha of 0.05 and 90% power, 1900 participants will be enrolled. The secondary neurological outcome will be poor functional outcome (modified Rankin scale 4–6) at 180 days after cardiac arrest. In this paper, a detailed statistical analysis plan is presented, including a comprehensive description of the statistical analyses, handling of missing data, and assessments of underlying statistical assumptions. Final analyses will be conducted independently by two qualified statisticians following the present plan.

**Discussion:**

This SAP, which was prepared before completion of enrolment, should increase the validity of the TTM trial by mitigation of analysis-bias.

## Background

The Targeted Hypothermia Versus Targeted Normothermia After Out-of-hospital Cardiac Arrest (TTM2 trial) is a continuation of the collaboration that resulted in the Target Temperature Management after out-of-hospital cardiac arrest trial (TTM trial) [1].

The TTM trial (NCT01020916) [1] was a multicentre, multinational, outcome assessor-blinded, parallel group, randomised clinical trial comparing two target temperature regimens of 33 °C and 36 °C in unconscious patients who had sustained return of spontaneous circulation after out-of-hospital cardiac arrest [1]. The trial did not demonstrate any significant difference in mortality rates or intact neurological survival between the two groups. Recently, the Therapeutic Hypothermia after Cardiac Arrest in Nonshockable Rhythm (HYPERION) trial was published [2]. This trial showed that among patients with coma who had been resuscitated from cardiac arrest with nonshockable rhythm, moderate therapeutic hypothermia at 33 °C for 24 h compared with targeted normothermia led to a higher percentage of patients who survived with a favourable neurologic outcome at day 90 (*P* = 0.04) [2].

The TTM2 trial is an international, multicentre, parallel group, investigator-initiated, randomised, superiority trial in which TTM with a target temperature of 33 °C after out-of-hospital cardiac arrest of a presumed cardiac or unknown cause will be compared with early treatment of fever (≥ 37.8 °C).

This publication will describe the statistical analyses of the primary and secondary outcomes in the TTM2 trial.

## Methods

The design of the TTM2 trial has been described in detail previously [3]. In short, the trial population will be adults (18 years of age or older) who experience a non-traumatic out-of-hospital cardiac arrest of a presumed cardiac or unknown cause with return of spontaneous circulation (ROSC). Patients will be eligible for enrolment if they meet all of the following inclusion criteria and none of the exclusion criteria.

### Inclusion criteria

1. Out-of-hospital cardiac arrest of a presumed cardiac or unknown cause
2. Sustained ROSC—defined as 20 min with signs of circulation without the need for chest compressions
3. Unconsciousness after sustained ROSC defined as FOUR score motor response < 4 and not able to obey verbal commands
4. Eligible for intensive care without restrictions or limitations
5. Screening for inclusion in the trial must be commenced no later than 180 min after ROSC

### Exclusion criteria

1. Unwitnessed cardiac arrest with an initial rhythm of asystole
2. Temperature on admission < 30 °C
3. On extracorporeal membrane oxygenation (ECMO) prior to ROSC
4. Suspected pregnancy
5. Intracranial bleeding
6. Severe chronic obstructive pulmonary disorder with long-term home oxygen therapy

### Co-enrolment with the TAME trial

The Targeted Therapeutic Mild Hypercapnia After Resuscitated Cardiac Arrest: A Phase III Multi-Centre Randomised Controlled Trial (TAME trial) (ACTRN12617000036314p) aims to determine whether targeted therapeutic mild hypercapnia improves neurological outcome at six months compared with standard care and targeted normocapnia. At certain sites, all TTM2 participants will also be enrolled in the TAME trial. We consider co-enrolment in TTM2 and TAME as an effective use of research resources. Adequate randomisation and a sample size as large as ours should lead to similar proportions of participants treated with targeted therapeutic mild hypercapnia in each of the TTM2 intervention groups. If there are no interactions between the TTM2 trial interventions and the TAME trial interventions, any beneficial or harmful effects of the TAME trial interventions will balance out.

An interaction between the TTM trial interventions and the TAME trial interventions is not likely. Theoretically, the TTM2 trial interventions are believed to have neuroprotective effects including reductions in metabolic rate and pathologic cell signalling, while the TAME trial interventions are believed to affect cerebral blood flow. Furthermore, we have studied the interaction between PaCO2 and temperature in the TTM trial and there was no statistically significant interaction (*P*_interaction_ = 0.95) [4]. If we show significant interactions, this will be handled as described under the ‘Assessments of underlying statistical assumptions’ section.

### Randomisation and blinding

Randomisation will be performed by an investigator in the emergency department, in the angiography unit, or in the intensive care unit via web-based application using permuted blocks with varying block sizes, stratified by site and co-enrolment in the TAME trial (no co-enrolment, TAME intervention arm 1, TAME intervention arm 2). Due to the nature of the intervention, the treating providers will not be blinded to the intervention. However, the outcome assessors, the prognosticators, the statisticians, the data managers, and the authors of the first version of the manuscript will be blinded to treatment allocation.

### Trial interventions

The intervention period for both intervention groups will be 40 h and commence at the time of randomisation. Rapid cooling in the hypothermia group will be achieved by means of cold fluids and state-of-the-art cooling devices, i.e. intravascular/body-surface/nasal/oesophageal cooling (physical cooling). A feedback-controlled system will be used to maintain the target temperature. In the normothermia arm, the aim will be early treatment of fever (≥ 37.8 °C) using pharmacological measures and physical cooling when needed (up to 72 h). For participants who develop a temperature of 37.8 °C (trigger), a device will be used and set at 37.5 °C. All participants will be sedated, mechanically ventilated, and haemodynamically supported throughout the intervention period. Participants who are managed at 33 °C will begin rewarming 28 h after randomisation.

Participants who remain unconscious will be assessed according to a conservative protocol based on the European Resuscitation Council (ERC)’s recommendations for neurological prognostication after cardiac arrest [3].

The main results of the trial will be published following the 6-month follow-up, results from the long-term follow-up and the outcome assessment of neurocognitive function will be presented separately [5].

### Outcomes

The outcomes were defined as primary and secondary [3]. The sample size was based on the primary outcome and our primary conclusions will be based on the results of the primary outcome. We ranked the outcomes in our outcome hierarchy according to clinical relevance and estimated the power of each outcome to ensure that we had sufficient power to confirm or reject the anticipated intervention effects [6].

#### Primary outcome

- All-cause mortality (dichotomous outcome)

#### Secondary outcomes

- Proportion of participants with a poor functional outcome (modified Rankin scale 4–6) (dichotomous outcome) [7], we will in a secondary analysis analyse the ordinal modified Rankin scale data (ordinal data)
- Number of days alive after hospital discharge within 6 months after randomisation (count data)
- Health-related quality of life using EQ5D-5 L (VAS) [8] (continuous outcome)
- Time-to-death (survival data) for each participant from randomisation until 6 months after the last participant is randomised. If death has not occurred, participants will be censored at this point

Dichotomous and continuous outcomes will be assessed at 30 days, 6 months, and 24 months after randomisation. For primary and secondary analyses, only the 6 months time point will be used.

### Sample size and power estimations

Based on the results of the previous TTM trial [1] and information in the International Cardiac Arrest Registry (INTCAR), we anticipate a mortality of 55% in the normothermia group [9]. Using an absolute risk reduction of 7.5% as anticipated intervention effect, an acceptable risk of type I error of 5%, and an acceptable risk of type II error of 10%, a total of 1862 (931 participants in each group) participants are required. This anticipated intervention effect corresponds to a relative risk reduction (RRR) of 13.6% and a number needed to treat (NNT) of 14 [10, 11]. Only 4/939 patients withdrew consent in TTM trial, and there were no missing data on mortality [1]. To allow for a possible loss to follow-up, we will recruit a total of 1900 participants.

We also estimated the statistical power of all secondary outcomes [6]. With an estimated sample size of 931 participants per group, the functional outcome measure (dichotomised mRS) has a power of 90% to detect a relative risk of 0.86 for a poor outcome (mRS 4–6) in 55% of cases in the control group. For the secondary outcome time-to-death, we estimate a power of > 90% based on the survival estimates mentioned above. We estimate a power of approximately 90% to detect a difference in 5 points on the EQ5D-5 L VAS-scale, based on a mean value of 70 in the control group and a standard deviation of 25 points [1, 3]. For the secondary outcome ‘days alive outside hospital’, we estimate a power of approximately 83%, based on simulations [3].

### General analysis principles

All analyses will be conducted according to the intention-to-treat principle (ITT), i.e. all randomised participants will be included in the analysis. A per protocol analysis will be performed if the number of participants in whom temperature management is withheld due to palliative care, early death or other reasons during the first six hours after randomisation exceeds 5% of the total trial population.

We will both assess if the thresholds for statistical significance and clinical significance are crossed (Bayes factor calculations will be reported in supplementary material) [12]. Assessment of clinical significance will be based on the anticipated intervention effects used in the sample size/power estimations [12]. Our primary conclusion will be based on the primary outcome, so all tests of statistical significance (including subgroup analyses) will be two-sided with a type I error risk of 5% [12].

It is generally acknowledged that regression analyses ought to be adjusted for the stratification variables used in the randomisation [13–15]. The TTM2 trial uses two stratification variables in the randomisation, i.e. ‘site’ and ‘co-enrolment in the TAME trial’ (no co-enrolment, TAME intervention arm 1, TAME intervention arm 2). We will primarily adjust all regression analyses for ‘site’ and ‘co-enrolment in the TAME trial’ to balance prognostic baseline characteristics across TTM2 trial intervention groups. We will also assess whether there are significant interactions between TTM2 trial interventions and the stratification variables (see the ‘Assessments of underlying statistical assumptions’ sections).

We will also perform the following subgroup analyses: sex (male compared to female), first presenting cardiac rhythm (shockable compared to non-shockable), presence of shock on admission (no shock on admission compared to shock on admission), age (at or above the median compared to below the median), and duration of cardiac arrest (at or above the median compared to below the median). We will present the results in forest plots.

### Statistical analyses

#### Analysis of dichotomous data

Dichotomised outcomes will be presented as proportions of participants in each group with the event, as well as risk ratios with 95% confidence intervals. Dichotomous outcomes will be analysed using mixed effects generalised linear models using a log link function with ‘site’ as a random intercept using an exchangeable covariance matrix, and co-enrolment will be included as a fixed effect.

#### Analysis of continuous data

Continuous outcomes will be presented as means and standard deviations for each group along with 95% confidence interval for the means of the groups and the mean differences between the groups. Continuous outcomes will be analysed using mixed effects linear regression with ‘site’ as a random intercept using an exchangeable covariance matrix, and co-enrolment will be included as fixed effect. We expect that a large proportion of the participants will die before assessment of quality of life. When assessing health-related quality of life, we will therefore in the primary analysis impute a ‘0’ for all participants who died or who are incapacitated and did not participate in the quality of life assessment. In a secondary analysis of quality of life, we will only include survivors at 6 months.

#### Analysis of count data

Count data will be presented as means, mean differences, and 95% confidence intervals or medians, interquartile ranges, and 95% confidence intervals (bootstrapping) depending on the observed distribution. Count data will be analysed by the van Elteren test stratified by ‘site’ [16].

#### Analysis of survival data

Survival data will be presented as median survival time, frequencies, and percentages per group as well as hazard ratios with 95% CIs. Survival data will be analysed using Cox regression adjusted for site and co-enrolment. We plan to present Kaplan-Maier curves.

### Handling of missing data

All randomised participants will be included in the primary analysis of all outcomes except in the primary analysis of health-related quality of life (please see the ‘Analysis of continuous data’ section). We anticipate that the proportion of missing values on primary and secondary outcomes will be less than 5%. However, we will in a secondary analysis consider using multiple imputation and present best-worst and worst best case scenarios if it is not valid to ignore missing data [17]. Best-worst and worst-best case scenarios assess the potential range of impact of the missing data for the trial results [17]. In the ‘best-worst’ case scenario, it is assumed that all patients lost to follow-up in the hypothermia group have had a beneficial outcome (have survived, had no poor functional outcome, and so forth), and all those with missing outcomes in the control group have had a harmful outcome (have not survived, have had poor functional outcome, and so forth) [17]. Conversely, in the ‘worst- best’ case scenario, it is assumed that all patients who were lost to follow-up in the experimental group have had a harmful outcome and that all those lost to follow- up in the control group have had a beneficial outcome [17]. When continuous outcomes are used, a ‘beneficial outcome’ will be defined as the group mean plus two SDs of the group mean (fixed imputation), and a ‘harmful outcome’ will be defined as the group mean minus two SDs of the group mean (fixed imputation) [17].

### Assessments of underlying statistical assumptions

We will systematically assess underlying statistical assumptions for all statistical analyses [18, 19]. For *all* regression analyses, both primary and secondary, we will test for major interactions between each covariate and the intervention variable. When assessing for major interactions, we will, in turn, include each possible first order interaction between included covariates and the intervention variable. For each combination, we will test if the interaction term is significant and assess the effect size. We will only consider that there is evidence of an interaction if the interaction is statistically significant after Bonferroni adjusted thresholds (0.05 divided by number of possible interactions (treatment variable interaction with ‘site’ and treatment variable interaction with ‘co-enrolment in the TAME trial’ = 0.025) and if the interaction shows a clinically important effect. If it is concluded that the interaction is significant, we will consider both presenting an analysis separately for each (e.g. for each site if there is significant interaction between the trial intervention and ‘site’) and an overall analysis including the interaction term in the model [18, 19].

#### Assessments of underlying statistical assumptions for dichotomous outcomes

We will assess if the deviance divided by the degrees of freedom is significantly larger than 1 to assess for relevant overdispersion. Overdispersion is the presence of greater variability (statistical dispersion) in a data set than would be expected based on a given statistical model, and this case considered using a maximum likelihood estimate of the dispersion parameter. To avoid analytical problems with either zero events or problems with all participants dying at a given site, we have only included sites planning to randomise a sufficient number of participants. However, we cannot exclude the risk that some sites might have problems with recruitment. We will, by checking if the number of participants is larger than 10 (rule of thumb) per site, consider pooling the data from small sites if the number of participants is too low [19].

#### Assessments of underlying statistical assumptions for linear regression

We will visually inspect quantile-quantile plots of the residuals [20, 21] to assess if the residuals are normally distributed and use residuals plotted against covariates and fitted values [20, 21] to assess for homogeneity of variances. If the plots show deviations from the model assumptions, we will consider transforming the outcome, e.g. using log transformation or square root and/or use robust standard errors [19–21].

#### Assessments of underlying statistical assumptions for Cox regression

We will visually inspect log-log plots stratified by treatment and adjusted for the effects of all covariates (continuous and categorical) [20, 22] to asses if the assumption of proportional hazards between the compared intervention groups is fulfilled. If the assumption of proportional hazards seems violated, we will consider using a non-parametric test (e.g. log rank test) or split the observation period into two (or more) separate observation periods [19].

### Statistical reports

Blinded data on all outcomes will be analysed by two independent statisticians [19]. Two independent statistical reports will be sent to the chief principal investigator and will be shared with the steering group and author group, and if there are discrepancies between the two primary statistical reports, then possible reasons for that will be identified and the steering group will decide which is the most correct result. A final statistical report will be prepared, and all three statistical reports will be published as supplementary material [19].

Mock tables are presented in Mock Tables TTM2.

## Discussion

The primary aim of this present publication is to minimise the risks of outcome reporting bias and erroneous data-driven results. We therefore present a pre-defined description of the statistical analysis plan for the TTM2 trial.

### Strengths

Our methodology has several strengths as it is pre-defined and we have limited problems with multiplicity because we only assess one primary outcome and our conclusions will primarily be based on the results of the primary outcome [12]. Our chosen outcomes are all patient-centred. Our primary outcome, all-cause mortality, remains perhaps the most reliable and patient-centred outcome and we assess all-cause mortality as a dichotomous outcome at one time point, which simplifies both the statistical methodology and the clinical interpretability, i.e. it is intuitively easy to assess whether a shown difference (effect size) is clinically important when comparing two proportions at one time point. We will analyse data in accordance to the intention-to-treat principle and, if necessary, use multiple imputation and best-worst/worst-best case scenarios to assess the potential impact of the missing data on the results [17]. Furthermore, we plan to systematically assess whether underlying statistical assumptions are fulfilled for all statistical analyses.

### Limitations

A potential limitation of the TTM2 trial are the potential heterogeneous intervention effects depending on the mode of cooling at different clinical sites, and the potential biased impact on the trial results if a large proportion of the randomised participants withdraw consent after regaining capacity. Another potential limitation is the planned co-enrolment with the TAME Trial; our results will be difficult to interpret if there are significant interactions between the TTM2 and TAME trial interventions. As mentioned (see the ‘Co-enrolment with the TAME trial’ section), we have studied the interaction between PaCO_2_ and temperature in the TTM trial and found no statistically significant interaction (*P*_interaction_ = 0.95) [4], and if we show significant interactions, this will be handled (see the ‘Assessments of underlying statistical assumptions’ section). Co-enrolment with the TAME trial also made it possible to increase the planned sample size from 1200 to 1900 participants.

We only assess one primary outcome and our primary conclusions will be based on the result of the primary outcome, but we assess several secondary outcomes, exploratory outcomes, and subgroup analyses which increase the risks of type I errors. It is a limitation that we do not adjust our thresholds for significance according to the number of outcome comparisons. Furthermore, our anticipated intervention effects used in the sample size estimation and the power estimations for the secondary outcomes are not based on previous valid studies because we have not identified such studies. We have pragmatically chosen these anticipated intervention effects based on clinical judgement and previous trial results [1, 2]. This increased risk of type I errors and the uncertainty regarding the anticipated intervention effects need to be considered when interpreting our trial results.

## Conclusion

We present a pre-defined description of the statistical analysis for the TTM2 trial. The risks of outcome reporting bias and erroneous data-driven results will be minimised if this statistical analysis plan is followed.

## Supplementary information

**Additional file 1.**

## Data Availability

All relevant data are available.
