# Supplementary material for: Targeted hypothermia versus targeted normothermia after out-of-hospital cardiac arrest: a statistical analysis plan
Source: Trials. 2020 Oct 7;21:831. doi: 10.1186/s13063-020-04654-y (PMC7542893; doi:10.1186/s13063-020-04654-y)
Supplement: Supplementary file 1 — Additional file 1. [file 13063_2020_4654_MOESM1_ESM.docx]

Mock tables TTM2

*Tables and figures planned for publication in main article/appendix of the trial, before results and review process.*

**Figure 1**


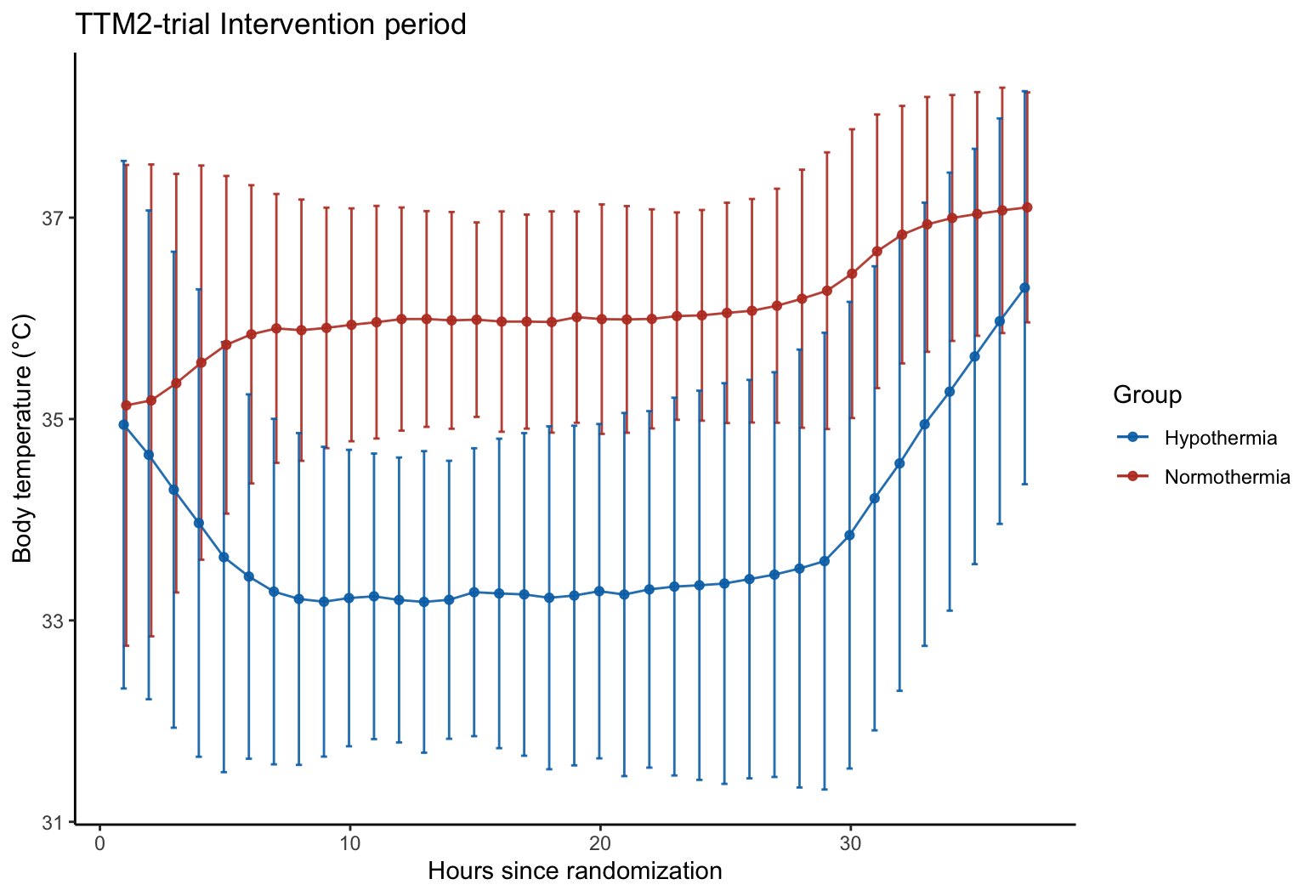


Body-temperature curves in the hypothermia and normothermia groups for the patients in whom temperature was recorded. The temperature curves display the means, and the I bars indicate ±2 SD (95% of the observations are within the error bars).

**Figure data from the TTM-trial:* Nielsen, N., J. Wetterslev, T. Cronberg, D. Erlinge, Y. Gasche, C. Hassager, J. Horn, J. Hovdenes, J. Kjaergaard, M. Kuiper, T. Pellis, P. Stammet, M. Wanscher, M. P. Wise, A. Åneman, N. Al-Subaie, S. Boesgaard, J. Bro-Jeppesen, I. Brunetti, J. F. Bugge, C. D. Hingston, N. P. Juffermans, M. Koopmans, L. Køber, J. Langørgen, G. Lilja, J. E. Møller, M. Rundgren, C. Rylander, O. Smid, C. Werer, P. Winkel and H. Friberg (2013). "Targeted temperature management at 33°C versus 36°C after cardiac arrest." N Engl J Med **369**.

Abbreviations: SD = standard deviation

**Figure 2**


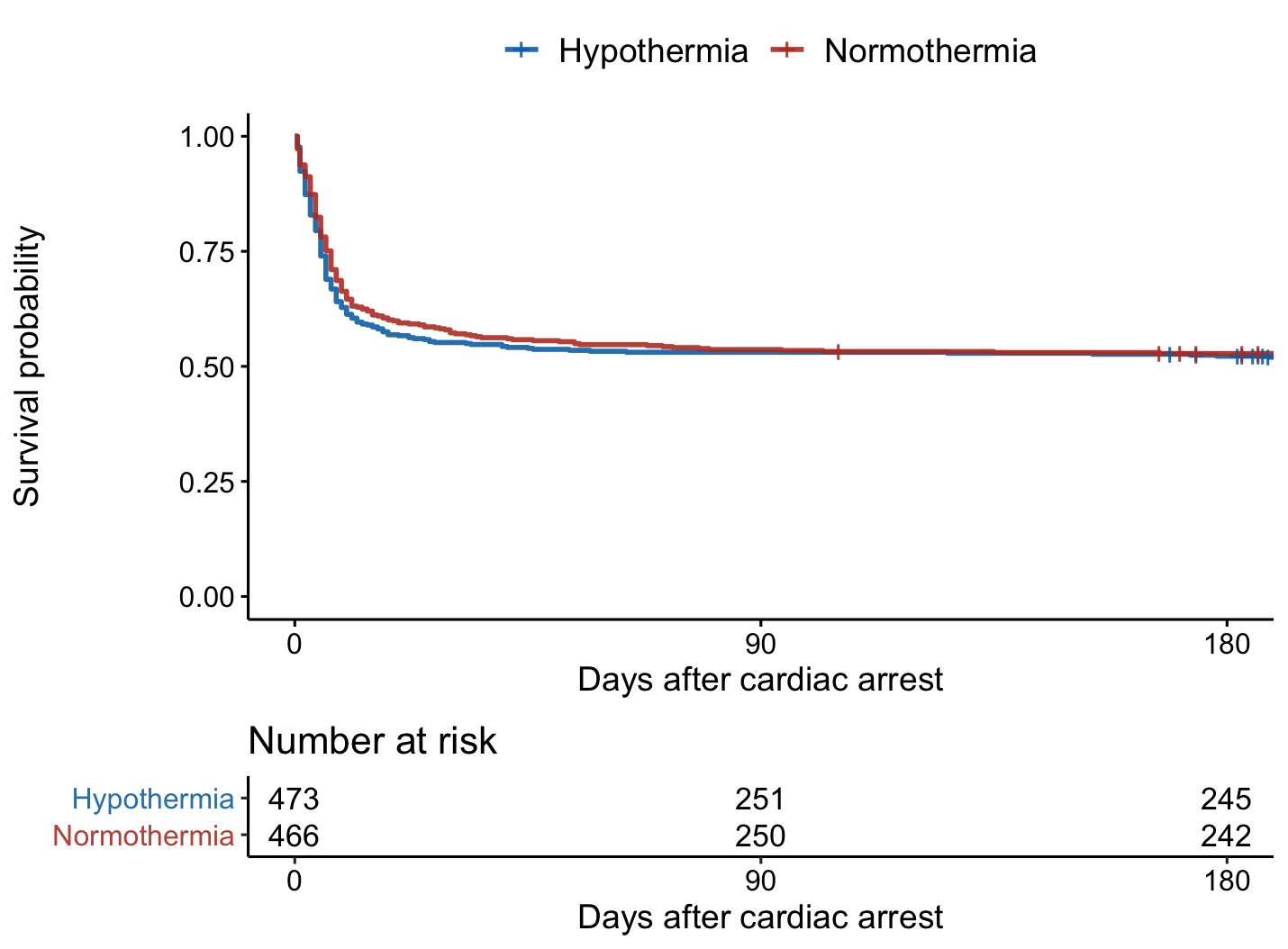


Kaplan–Meier estimates of the probability of survival for patients assigned to hypothermia or normothermia and the number of patients at risk at each time point. P-value 0.51. The p-value is derived by stratified Cox-regression.

**Figure data from the TTM-trial:* Nielsen, N., J. Wetterslev, T. Cronberg, D. Erlinge, Y. Gasche, C. Hassager, J. Horn, J. Hovdenes, J. Kjaergaard, M. Kuiper, T. Pellis, P. Stammet, M. Wanscher, M. P. Wise, A. Åneman, N. Al-Subaie, S. Boesgaard, J. Bro-Jeppesen, I. Brunetti, J. F. Bugge, C. D. Hingston, N. P. Juffermans, M. Koopmans, L. Køber, J. Langørgen, G. Lilja, J. E. Møller, M. Rundgren, C. Rylander, O. Smid, C. Werer, P. Winkel and H. Friberg (2013). "Targeted temperature management at 33°C versus 36°C after cardiac arrest." N Engl J Med **369**.

**Figure 3**


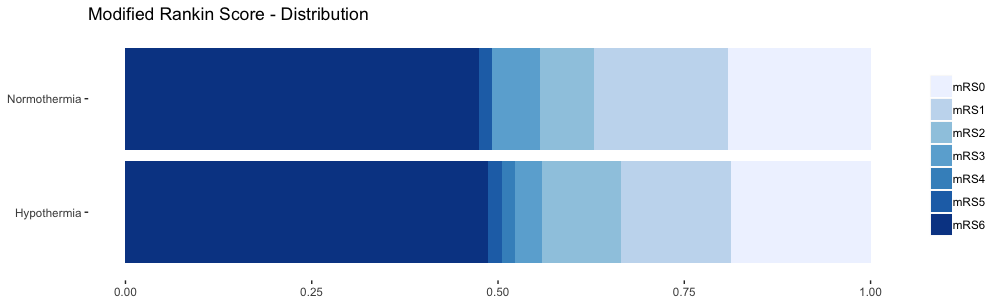


The distribution of Modified Rankin Scores (mRS) at 6 months after randomization. Scores on the modified Rankin scale range from 0 to 6, with 0 representing no symptoms, 1 no clinically significant disability, 2 slight disability, 3 moderate disability, 4 moderately severe disability, 5 severe disability, and 6 death. Patients who were lost to follow-up are not included.

** mRS-scores*

**Figure data from the TTM-trial:* Nielsen, N., J. Wetterslev, T. Cronberg, D. Erlinge, Y. Gasche, C. Hassager, J. Horn, J. Hovdenes, J. Kjaergaard, M. Kuiper, T. Pellis, P. Stammet, M. Wanscher, M. P. Wise, A. Åneman, N. Al-Subaie, S. Boesgaard, J. Bro-Jeppesen, I. Brunetti, J. F. Bugge, C. D. Hingston, N. P. Juffermans, M. Koopmans, L. Køber, J. Langørgen, G. Lilja, J. E. Møller, M. Rundgren, C. Rylander, O. Smid, C. Werer, P. Winkel and H. Friberg (2013). "Targeted temperature management at 33°C versus 36°C after cardiac arrest." N Engl J Med **369**.

**Figure 5**

Risk ratio for death at 180 days, according to subgroup. Risk ratios are derived from a stratified generalised linear model with site as a random intercept. The forest plot shows the risk ratios for six predefined subgroups. The horizontal bars represent 95% confidence intervals. The events are the total events six months after randomization. P values are for the tests of subgroup heterogeneity (tests of interactions). ROSC denotes return of spontaneous circulation. For unwitnessed cardiac arrests the time to ROSC was calculated form time of emergency call. Shock at admission was defined as a systolic blood pressure <90mmHg for >30min or end-organ hypoperfusion (cool extremities, urine output <30mm/hour, heart rate <60 beats/minute)


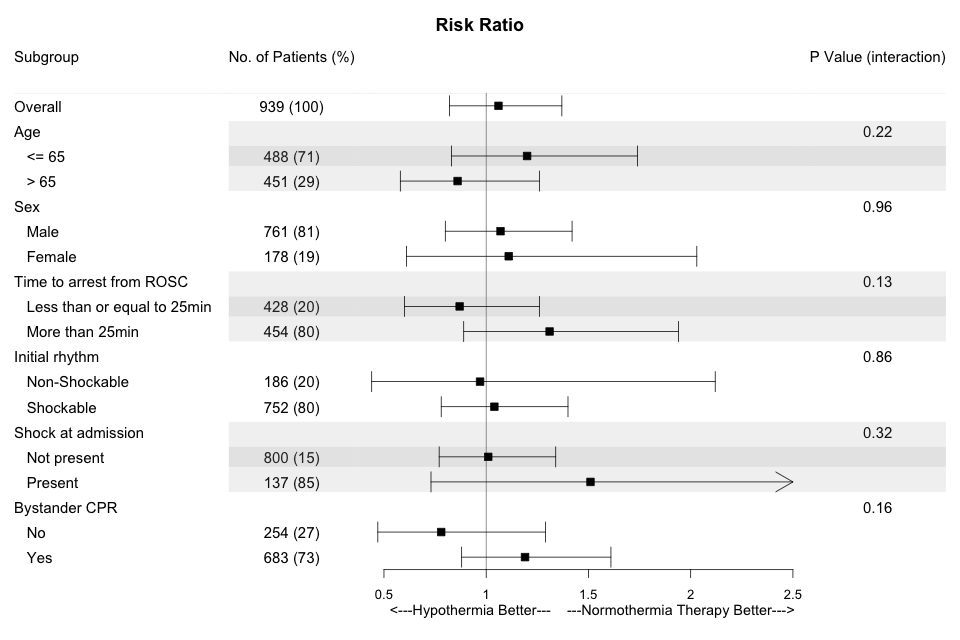


** Risk ratios*

**Figure data from the TTM-trial:* Nielsen, N., J. Wetterslev, T. Cronberg, D. Erlinge, Y. Gasche, C. Hassager, J. Horn, J. Hovdenes, J. Kjaergaard, M. Kuiper, T. Pellis, P. Stammet, M. Wanscher, M. P. Wise, A. Åneman, N. Al-Subaie, S. Boesgaard, J. Bro-Jeppesen, I. Brunetti, J. F. Bugge, C. D. Hingston, N. P. Juffermans, M. Koopmans, L. Køber, J. Langørgen, G. Lilja, J. E. Møller, M. Rundgren, C. Rylander, O. Smid, C. Werer, P. Winkel and H. Friberg (2013). "Targeted temperature management at 33°C versus 36°C after cardiac arrest." N Engl J Med **369**.

**Figure 4**

Consort flow chart.

Screened for eligibility (n= )

## Follow-Up

Allocated to intervention (n= )

♦ Received allocated intervention (n= )

♦ Did not receive allocated intervention (give reasons) (n= )

## Allocation

Allocated to intervention (n= )

♦ Received allocated intervention (n= )

♦ Did not receive allocated intervention (give reasons) (n= )

Randomized (n= )

Excluded (n= )

♦  Not meeting inclusion criteria (n= )

♦  Other reasons (n= )

Specify and group reasons

Lost to follow-up (give reasons) (n= )

Discontinued intervention (give reasons) (n= )

Included in analysis of mortality ( n= )

Included in analysis of neurological function ( n= )

Included in per-protocol analysis if applicable (n=)

Lost to follow-up (give reasons) (n= )

Discontinued intervention (give reasons) (n= )
Included in analysis of mortality ( n= )

Included in analysis of neurological function ( n= )

Included in per-protocol analysis if applicable (n=)

**Table 1**

Characteristics of the trial population

| Characteristic | Hypothermia  (n=950) | Normothermia (n=950) |
| --- | --- | --- |
| Age – years (SD) | 65 (12) | 65 (12) |
| Male sex – no. (%) | 760 (80) | 760 (80) |
| *Medical History* |  |  |
| Previous hypertension - no. (%) | 760 (80) | 760 (80) |
| Previous diabetes - no. (%) | 760 (80) | 760 (80) |
| Previous myocardial Infarction - no. (%) | 760 (80) | 760 (80) |
| Previous PCI – no. (%) | 760 (80) | 760 (80) |
| Previous coronary artery bypass grafting – no. (%) | 760 (80) | 760 (80) |
| Previous heart failure - no.(%) | 760 (80) | 760 (80) |
| NYHA III or IV – no. (%) | 760 (80) | 760 (80) |
| Charlson Comorbidity index – median (IQR) | 40 (30-30) | 40 (30-30) |
| *Characteristics of the cardiac arrest* |  |  |
| Location of arrest |  |  |
| - Home – no. (%) | 760 (80) | 760 (80) |
| - Public place – no. (%) | 76 (10) | 76 (10) |
| - Other – no. (%) | 76 (10) | 76 (10) |
| Bystander witnessed arrest – no. (%) | 760 (80) | 760 (80) |
| Bystander CPR performed – no. (%) | 760 (80) | 760 (80) |
| First monitored rhythm |  |  |
| Shockable rhythm – no. (%) | 760 (80) | 760 (80) |
| - Ventricular fibrillation no. (%) | 760 (80) | 760 (80) |
| - Non-perfusing ventricular tachycardia no. (%) | 760 (80) | 760 (80) |
| - ROSC after bystander defibrillation no. (%) | 760 (80) | 760 (80) |
| - Unknown rhythm – shock administered no. (%) |  |  |
| Non-shockable rhythm | 760 (80) | 760 (80) |
| - Pulseless electrical activity - no. (%) | 760 (80) | 760 (80) |
| - Asystole no. - (%) | 760 (80) | 760 (80) |
| - Unknown – no shock administered - no. (%) | 760 (80) | 760 (80) |
| Time from cardiac arrest to sustained return of spontaneous circulation – minutes (IQR) | 20 (18-40) | 20 (18-40) |
| Time from arrest to randomization – minutes (IQR) | 20 (18-40) | 20 (18-40) |
| *Clinical characteristics on admission* |  |  |
| First measured body temperature - °C (SD) | 35.0°C (0.5) | 35.0°C (0.5) |
| FOUR motor score – median (IQR) | 40 (30-30) | 40 (30-30) |
| Corneal reflexes bilaterally present - no. (%) | 760 (80) | 760 (80) |
| Pupillary reflexes bilaterally present - no. (%) | 760 (80) | 760 (80) |
| Serum pH – pH (SD) | 6.7 (0.4) | 6.7 (0.4) |
| Serum lactate - mmol/L (SD) | 6.7 (4.5) | 6.7 (4.5) |
| Shock on admission - no. (%) | 760 (80) | 760 (80) |
| ST-elevation myocardial infarction - no. (%) | 760 (80) | 760 (80) |
|  |  |  |

PCI: Percutaneous coronary intervention, CPR: Cardiopulmonary resuscitation, NYHA: New York Heart Association class for heart failure, ROSC: Return of spontaneous circulation, FOUR: Full Outline of UnResponsiveness score for com.

**Table data are fictious*

*Abbreviations:*

*SD= standard deviation*

*No. = number*

*PCI = percutaneous coronary intervention*

*NYHA= New York Heart Association:*

*IQR=interquartile range*

*CPR = Cardiopulmonary resuscitation*

*ROSC = Return of spontaneous circulation*

**Table 2**

| **Table XX Primary and secondary outcome results** | | | |
| --- | --- | --- | --- |
|  | Targeted hypothermia group (n= XX) | Targeted normothermia group (n= XX) | Estimate (95% CI) |
| **Primary outcome** |  |  |  |
| All-cause mortality (proportion (%)) | XX/ YY (ZZ %) | XX/ YY (ZZ %) | Relative risk XX (YY to ZZ); P value ZZ |
| **Secondary outcomes** |  |  |  |
| Poor functional outcome (modified Rankin scale 4-6) (proportion (%)) | XX/ YY (ZZ %) | XX/ YY (ZZ %) | Relative risk XX (YY to ZZ) |
| Number of days alive after hospital discharge within 6 months (mean) | XX | XX | Mean difference XX (YY to ZZ) |
| Health-related quality of life using EQ5D-5L (VAS) (mean) | XX | XX | Mean difference XX (YY to ZZ) |
| Time-to-death (median survial time) | XX | XX | Hazard ratio XX (YY to ZZ) |

*A P value will be presented for the principal analysis of the primary outcome only*

*All analyses, except number of days alive after hospital discharge, were adjusted for ‘site’ and ‘co-enrolment in the TAME. Number of days alive after hospital discharge was only adjusted for ‘site’*

*Abbreviations: CI = confidence interval*

**Table 3**

In-hospital characteristics

| Characteristic | Hypothermia  (n=950) | Normothermia (n=950) |
| --- | --- | --- |
| *Cardiac Interventions* | 760 (80) | 760 (80) |
| - Coronary angiogram performed | 760 (80) | 760 (80) |
| - Percutaneous coronary intervention | 760 (80) | 760 (80) |
| - Coronary artery bypass grafting performed | 76 (10) | 76 (10) |
| - Implantable cardiac defibrillator | 76 (10) | 76 (10) |
| *Serious Adverse Event – no. (%)* | 760 (80) | 760 (80) |
| Pneumonia | 760 (80) | 760 (80) |
| Bleeding | 760 (80) | 760 (80) |
| Arrhythmia resulting in hemodynamic compromise | 760 (80) | 760 (80) |
| - Bradycardia requiring pacing | 760 (80) | 760 (80) |
| Device-related skin complication | 760 (80) | 760 (80) |
| Sepsis | 760 (80) | 760 (80) |
| - Septic shock | 760 (80) | 760 (80) |
| Unexpected serious adverse event# | 760 (80) | 760 (80) |

#After independent adjudication

**Table data are fictious*
